# The Combination of Ibrutinib with BH3 Mimetics or Dichloroacetate Is Effective in B-CLL

**DOI:** 10.3390/cells14171343

**Published:** 2025-08-29

**Authors:** Joaquín Marco-Brualla, Oscar Gonzalo, Gemma Azaceta, Isabel Izquierdo, Luis Palomera, Martín Villalba, Isabel Marzo, Alberto Anel

**Affiliations:** 1 Apoptosis, Immunity and Cancer Group, Department of Biochemistry and Molecular and Cell Biology, Aragon Health Research Institute (IIS-Aragon), University of Zaragoza, 50009 Zaragoza, Spain; joaquin_marco_91@hotmail.com (J.M.-B.);; 2 Faculty of Pharmacy, University San Pablo-CEU, 28660 Boadilla del Monte, Spain; 3 Hematology Department, Lozano Blesa Clinical Hospital, 50009 Zaragoza, Spain; 4 IRMB, University of Montpellier, INSERM, CNRS, Montpellier CHU, 34295 Montpellier, France

**Keywords:** B-CLL, dichloroacetate, BH3 mimetics, ibrutinib, Bcl-XL, Mcl-1

## Abstract

Since its discovery, the BTK inhibitor ibrutinib has redefined the standard treatments for hematological cancers, such as chronic lymphocytic leukemia (CLL). However, concerns exist regarding its secondary effects in humans and its occasional lack of efficacy in certain malignancies. Therefore, combined therapies with ibrutinib have emerged as promising new approaches. In this study, we aimed to explore its therapeutic potential through different approaches. For this purpose, we combined this drug with the BH3 mimetics ABT-199 and ABT-737, which inhibit anti-apoptotic members of the Bcl-2 family, and with the PDK1 inhibitor dichloroacetate (DCA), respectively. As cell models, we used ex vivo samples from patients and also selected the in vitro CLL cell line Mec-1, generating two sub-lines overexpressing Bcl-XL and Mcl-1, a common feature in this cancer. Results demonstrated a synergistic effect for both approaches, in all tumor cells tested, for both cytostatic and cytotoxic effects. Mechanistically, the expression of Bcl-2-family proteins was explored, exhibiting increases in pro-apoptotic, but also in anti-apoptotic, proteins upon ibrutinib treatment and a relative increase in the amount of the pro-apoptotic protein PUMA after treatment with DCA. Our data provides new insights into combined therapies with ibrutinib for CLL, which further expands our knowledge and the potential of this drug for cancer treatment.

## 1. Introduction

Chronic lymphocytic leukemia (CLL) remains the most common type of leukemic malignancy [1]. It is characterized as an abnormal presence and accumulation of mature and transformed B-cells in blood (lymphocytosis), which can eventually colonize the spleen, lymph nodes and bone marrow. Tumor transformation is often triggered by previous chromosomal alterations, followed by other somatic mutations [2]. CLL may develop from monoclonal B-lymphocytosis (MBL), characterized by < 5000 lymphocytes per mm^3^ in blood and no effect in other organs, and small lymphocytic leukemia (SLL), featuring the same count condition but also involving spleen or lymph node infiltration. When the count surpasses 5000 cell per mm^3^, it can be considered CLL [3]. These mature tumor lymphocytes are unable to fulfill their regular immune function. As a consequence of this lowering in humoral immunity, frequent opportunistic infections occur, as well as other symptoms, such as: fatigue, weight loss, anemia and sudden fever. If not treated in time, further mutations may occur, turning leukemia cells more resistant over time [2].

One of these alterations is the overexpression of anti-apoptotic Bcl-2 family proteins [4]. Briefly, these proteins have Bcl-2 homology (BH) domains in common and can either promote or inhibit apoptosis, depending on the specific protein. Several of the anti-apoptotic proteins, such as Bcl-2 itself, Bcl-XL or Mcl-1, have consistently been found to be overexpressed in hematologic malignancies, such as CLL [4]. In contrast, Bcl-2 family proteins containing just the BH3 domain (BH3-only proteins) are exclusively pro-apoptotic. Most of them are sequestered and inhibited by the anti-apoptotic members of their family and released after a pro-apoptotic stimulus, thereby promoting cell death [5]. 

Notwithstanding the rise of cancer immunotherapy in the last decade, current B-CLL treatment options usually comprise anti-CD20 monoclonal antibodies (rituximab or obinutuzumab), BTK inhibitors and more recently the Bcl-2 inhibitor venetoclax or ABT-199 [5]. In particular, ibrutinib was the first and most widely known BTK inhibitor to be approved by the FDA for both first-line treatment and relapse CLL, following its promising clinical results [6,7]. It irreversibly and covalently binds BTK, thus dampening the B-cell receptor (BCR) signaling pathway, essential for B-cell survival and proliferation and usually overactivated in B-cell tumors [8]. Over recent years, ibrutinib has successfully improved the overall survival of patients, across different countries and at various stages of the malignancy, compared to other conventional treatments [9,10,11,12,13,14]. Nonetheless, despite its generally low toxicity profile, several secondary adverse effects have been reported [15] and its therapeutic role has even been questioned following its recent withdrawal in other malignancies [16]. Considering the fact that ibrutinib is still the most commonly used BTK inhibitor, especially in less wealthy countries, improvements in its use would be beneficial for a large number of patients.

A potential improvement would be to use lower doses of ibrutinib in combination therapies, with the hope of finding synergistic effects [17]. In fact, chemotherapy with this molecule and anti-CD20 monoclonal antibodies constitutes a gold standard of care for CLL. Another successful partner for ibrutinib could be the aforementioned Bcl-2 family protein inhibitors, especially venetoclax (or ABT-199). This BH3 mimetic efficiently inhibits the anti-apoptotic protein Bcl-2 and has been combined with ibrutinib in clinical trials against CLL with positive results [5,18]. Other BH3 mimetics, such as ABT-737 and navitoclax (ABT-263) (both Bcl-2 and Bcl-XL inhibitors), have been developed, with promising pre- and clinical results, respectively [19,20].

Another synergistic approach with ibrutinib would be the combination with metabolic drugs. Deregulation of cellular energetics is a well-established hallmark of cancer [21], characterized by aerobic glycolysis, or the Warburg effect, and CLL is no stranger to this feature [22]. Metabolic drugs, such as dichloroacetate (DCA), which limits glycolysis by forcing OXPHOS through PDK1 inhibition, have been proven to change the expression of target proteins, such as ICAM-1 or DR5, increasing the sensitivity of tumor cells to immunotherapy or to the death ligand TRAIL [23,24,25].

In this study, we sought to explore the synergistic potential of ibrutinib with different therapeutic approaches against several B-CLL cell models. Results demonstrate that DCA can sensitize Mec-1 cell lines to the cytostatic and cytotoxic action of ibrutinib, even in those overexpressing Bcl-XL or Mcl-1. Similarly, ABT-199 or ABT-737 also induced cell death and diminished cell growth after ibrutinib exposure. In addition, both ibrutinib combination approaches were also able to potently synergize against ex vivo CLL samples from patients.

## 2. Materials and Methods

### 2.1. Cell Lines, Cells from Patients and Cell Culture

B-CLL cell line Mec-1 was kindly provided by Dr. Dolors Colomer. Mec-1 cells overexpressing the anti-apoptotic proteins Bcl-XL (‘Mec-1 Bcl-XL’) or Mcl-1 (‘Mec-1 Mcl-1′) were generated by retrovirus transfection [26,27]. In brief, pBABE vectors containing either Bcl-XL or Mcl-1 genes were obtained. Afterwards, retroviral supernatants were isolated after incubation of their genetic material and pBABE vectors with 293T cells. Mec-1 cells were subsequently transfected and puromycin-resistant clones were selected. Finally, transfection efficiency was checked by the expression of each of those proteins by Western blot (see Results section). To simplify this study’s understanding, the standard, unmodified Mec-1 cell line is hereafter designated as ‘Mec-1 WT’ (for wild type).

Mec-1 cell lines were cultured in IMDM medium (Gibco, Waltham, MA, USA), while for 293T cells, DMEM (Gibco, Waltham, MA, USA) was used. For all of them, these media were supplemented with 10% heat-inactivated FBS (Sigma, St. Louis, MO, USA), a solution of penicillin–streptomycin (Pan Biotech, Aidenbach, Germany) and GlutaMAX (Gibco, Waltham, MA, USA). These supplemented RPMI and IMDM media will be named as ‘complete media’ from now on.

Blood samples from B-CLL patients were provided by Dr. Luis Palomera, Gemma Azaceta and Isabel Izquierdo (Immunology Department, Lozano Blesa Clinical Hospital, Zaragoza, Spain). The procedure was previously approved by the local Ethical Commission for Research from the Aragón Autonomous Region (CEICA), with permission numbers PI16/0129 and PI19/452. The procedure included approved consent from the patients and was in accordance with the Declaration of Helsinki. Peripheral blood mononuclear cells (PBMCs) were extracted by density gradient centrifugation using Ficoll-Paque^TM^ Plus (GE Healthcare, Chicago, IL, USA). Briefly, blood was prediluted 1:1 in PBS and carefully placed over a Ficoll-Paque^TM^ Plus-containing conical tube, also in a 1:1 proportion. Then, cells were centrifuged at 450× *g* for 20 min and the white ring containing the PBMCs was extracted and washed with PBS. Finally, cells were resuspended in complete RPMI, and cell count and viability were assessed by the trypan blue staining method in a hemocytometer before experimentation.

### 2.2. Cell Incubation with Drugs

Mec-1 WT, Mec-1 Bcl-XL and Mec-1 Mcl-1 cells were counted in a hemocytometer and seeded at a concentration of 5 × 10^5^ cells/mL in flat-bottomed 96-well plates in complete RPMI medium. For CLL ex vivo samples, 5 × 10^6^ cells/mL were seeded in similar plates, in complete RPMI medium, supplemented with human recombinant IL-4 (Preprotech, Cranbury, NJ, USA) at a concentration of 100 IU/mL. Next, tumor cells were incubated for specific times with the drugs indicated in the experiments, which were: ibrutinib (Selleckchem, Houston, TX, USA) at concentrations ranging from 0.78–25 μM, DCA (Sigma, St. Louis, MO, USA) at 10–20 mM and ABT-199 (MedChemExpress, Sollentuna, Sweden) and ABT-737 (MedChemExpress, Sollentuna, Sweden), both at 5–10 μM.

### 2.3. Flow Cytometry Assays

For the analysis of apoptosis induced by drugs, a FACSCalibur flow cytometer was used. Cells from each experimental point were incubated with Annexin-V-DY634 (Immunostep, Salamanca, Spain) in annexin binding buffer (140 mM NaCl, 2.5 mM CaCl_2_, 10 mM HEPES/NaOH, pH 7.4). Then, cells were washed with PBS and assessed in a FACSCalibur flow cytometer, using the CellQuestPro 6.0 software (BD Biosciences, Franklin Lakes, NJ, USA). Finally, further analysis was checked by Flow Jo v10.8.1 software (Tree Star Inc., San Francisco, CA, USA).

### 2.4. Cell Growth Assays

The modified Mosmann method [28] was employed for the proliferation assays of tumor cells. Cells were initially seeded in 96-well plates, as described above. After the corresponding incubation times with drugs, 10 μL of MTT solution (consisting of 5 mg/mL of 3-(4,5-dimethylthiazol-2-yl)-2,5 diphenyltetrazolium bromide, in PBS) was mixed in at every experimental point and incubated again for about 3 h at 37 °C. After formation of the purple formazan precipitate, 96-well plates were centrifuged at 450× *g* for 10 min, supernatant was carefully removed and crystals were resuspended and dissolved using an isopropanol/HCl (50 mM) solution. Next, absorbance at 550 nm was measured in a Dynatech MR5000 microplate reader (Dynex Technologies Inc.). Finally, results were analyzed, normalizing results by expressing untreated cell absorbance as 100% cell growth.

### 2.5. Western Blot Experiments

Cells were seeded at 3 × 10^5^ cells/mL in 6-well plates in complete RPMI medium. After incubation with their corresponding treatments, at least 3 × 10^6^ cells were collected for each experimental point. These cells were centrifuged (450× *g*, 5 min) and resuspended in a lysis buffer, containing: 10 mg/mL leupeptin, 1 mM PMSF, 1 mM EDTA, 150 mM NaCl, 10% glycerol and 1% Triton X-100 in 50 mM Tris/HCl (pH 7.4) and distilled water, at a proportion of 30 μL/10^6^ cells. Following 30 min of incubation at 4 °C, cell lysates were centrifuged in the same conditions to discard cell membranes. Then, proteins from these supernatants were separated by PAGE by mixing them in a proportion of 2:1 in electrophoresis loading buffer (3% *w*/*v* SDS, 300 μM Na_2_MoO_4_, 30 mM Na_4_P_2_O_7_, 30 mM NaF, 0.06% *v*/*v* bromophenol blue, 30% *v*/*v* β-mercaptoethanol, 30% *v*/*v* glycerol, 150 mM Tris/HCl), loading in SDS-12% polyacrylamide gels and applying electric current in the system. Separated proteins were transferred to nitrocellulose membranes and blocked with PBS-T buffer (5% *w*/*v* skimmed milk powder, 120 mM NaCl; 0.1% *v*/*v* Tween-20 and 10 mM Tris/HCl pH 8.0). Next, membranes were exposed to one of the following antibodies: anti-Bcl-2 (Abcam, Cambridge, UK), anti-Bcl-XL (Cell Signaling, Danvers, MA, USA), anti-Bim (Calbiochem, San Diego, CA, USA), anti-Mcl-1 (Santa Cruz Biotech, Dallas, TX, USA), anti-Noxa (Abcam, Cambridge, UK), anti-PUMA (Abcam, Cambridge, UK), anti-p-Bcl-2 (Millipore, Burlington, MA, USA), anti-p-Bcl-XL (Cell Signaling, Danvers, MA, USA) or anti-β-actin (Sigma, St. Louis, MO, USA). After several washes with PBS-T buffer, membranes were incubated with their corresponding peroxidase-labeled antibody (Sigma, St. Louis, MO, USA) and protein expression was evaluated by exposure of the membrane to Pierce ECL Western Blotting Substrate (Thermo Scientific, Waltham, MA, USA).

### 2.6. Immunoprecipitation

To evaluate the binding of PUMA with Bcl-2 or Bcl-XL, immunoprecipitation was performed. For this purpose, cell pellets were collected as described in this section and then protein extracts were obtained by incubating pellets with CHAPS buffer (2% *w*/*v* 3-[(3-cholamidopropyl)dimethylammonio]-1-propanesulfonate or CHAPS, 150 mM NaCl, protease inhibitors from Roche (Basel, Switzerland) and 10 mM HEPES pH 7.4) and precleaned with Sepharose^®^ CL-4B beads (Sigma, St. Louis, MO, USA). One aliquot of this step was saved and, afterwards, cell lysates were subjected to immunoprecipitation by incubation with an anti-PUMA antibody (Abcam, Cambridge, UK) overnight at 4 °C. Precipitation of the protein–antibody complexes was achieved by addition of agarose beads (Protein A/G PLUS-Agarose, Santa Cruz Biotech, Dallas, TX, USA) for 2 h at 4 °C and centrifugation at 300× *g* for 1 min. An aliquot of this supernatant (‘depleted’) was also saved and, then, agarose beads were washed three times with CHAPS buffer, mixed with electrophoresis loading buffer and proteins were released by heating at 70 °C for 10 min. Finally, samples were loaded in electrophoresis gels and further processed as mentioned in the previous subsection. Protein expression changes were assessed by densitometry, using the ImageJ 1.50i software (NIH, Bethesda, MD, USA). Results are expressed as the ‘x-fold change’ of treated vs. untreated cells.

### 2.7. Statistical Analysis

The statistical significance of obtained data was assessed by calculation of Student’s t-test for paired variants, using the software program GraphPad Prism 8.0 (GraphPad Software Inc., San Diego, CA, USA). For all cases, a comparison was declared significant when the *p* value was under 0.05.

## 3. Results

### 3.1. Generation of Sublines from Mec-1 Cells

**Figure 1:**
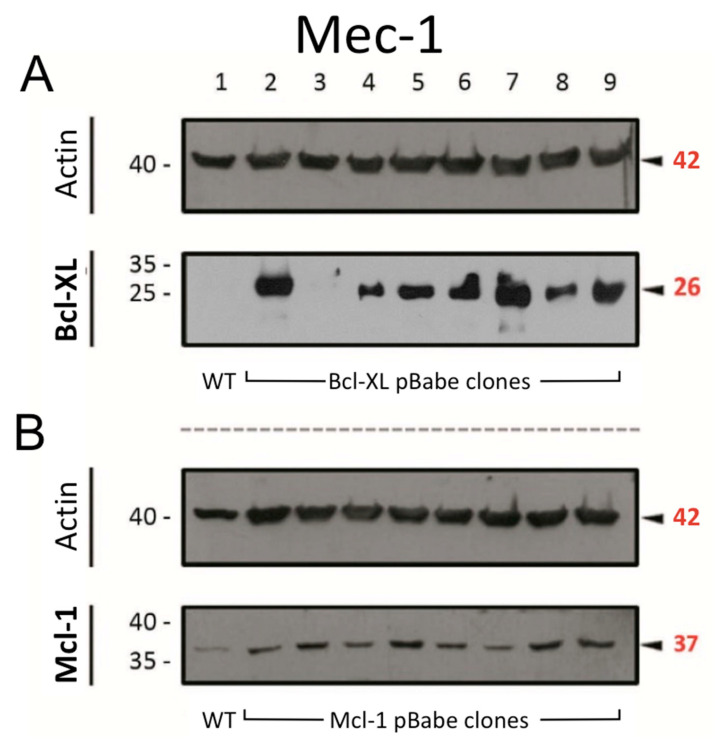
Protein expression of (**A**) Bcl-XL and (**B**) Mcl-1 in Mec-1 WT (lane 1) and transfected clones of this cell line (lanes 2–9) by Western blot. Actin was used as loading control. Numbers on the side of the immunoblots correspond to the molecular weight of the proteins expressed in kDa.

As mentioned in the Introduction, the overexpression of anti-apoptotic Bcl-2 family members is a common feature of B-CLL [4]. Therefore, prior to the drug combination experiments, we transfected Mec-1 cells to induce a higher expression of either Bcl-XL or Mcl-1, as described in the Materials and Methods. The results from these transfections are displayed in Figure 1. For both proteins, their expression increased in most of the generated clones, especially when compared with their actin loading controls. After consideration, clones 9 (for Bcl-XL) and 8 (for Mcl-1) were selected to establish the new sublines, named as ‘Mec-1 Bcl-XL’ and ‘Mec-1 Mcl-1′, respectively. As noted in the previous section, generic Mec-1 cells will be referred to as ‘Mec-1 WT’ from now on. 

### 3.2. Combination of BH3 Mimetics and Ibrutinib in Cell Growth and Apoptosis in Mec-1 Cell Lines

Once we obtained our B-CLL cell lines, we began by testing the combinatory effect of ibrutinib with the BH3 mimetics ABT-737 or ABT199. Firstly, we made a dose–response curve of ibrutinib +/− BH3 mimetics, at ibrutinib ranges of 0.78–25 μM and fixing the concentration of mimetics to 10 μM. 

**Figure 2:**
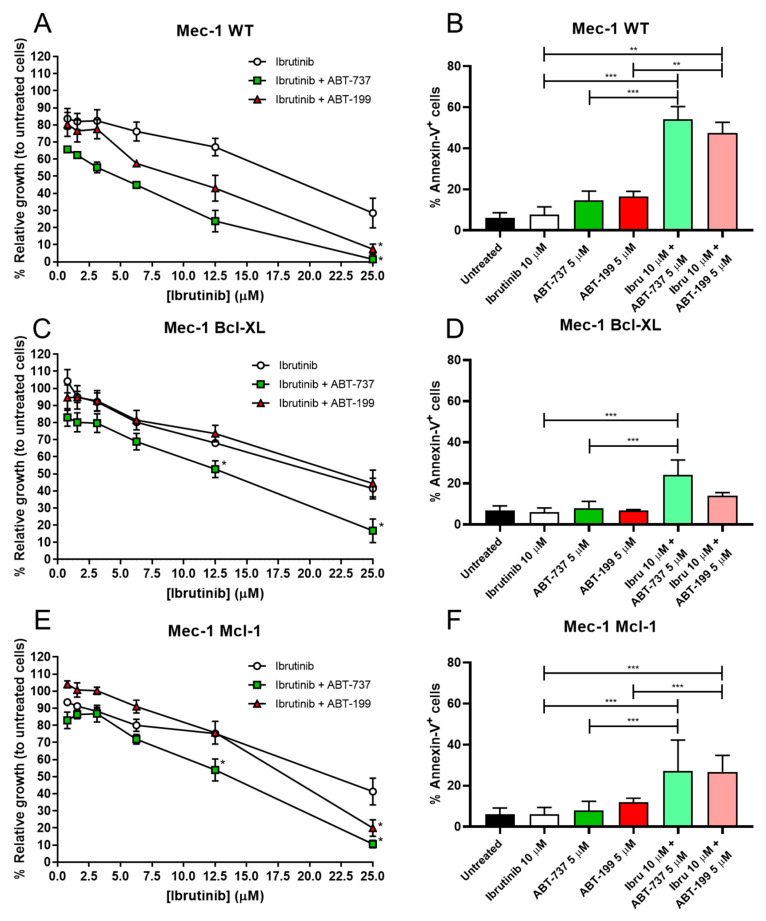
Effect of 24 h incubation of ibrutinib and/or ABT-737/ABT-199 on cell growth and apoptosis in Mec-1 WT (**A**,**B**) Mec-1 Bcl-XL (**C**,**D**) and Mec-1 Mcl-1 (**E**,**F**) cells. For cell growth assays (**A**,**C**,**E**), cells were incubated with ABT-737/ABT-199 (5 μM) and several doses of ibrutinib and MTT assays were then performed. For cell death evaluation, cells were incubated with ABT-737/ABT-199 (5 μM) and ibrutinib (10 μM) and flow cytometry (**B**,**D**,**F**) was performed. Data from MTT are expressed as the mean ± SD of percentage of relative growth, compared to untreated cells (100%); data from flow cytometry are reflected as the mean ± SD of percentage of annexin-V-positive cells. n = 3–6. ** *p* < 0.01; *** *p* < 0.001.

MTT assays revealed that ibrutinib alone affected all three cell lines equally, with very similar dose–response curves, obtaining IC50 values of 19 µM for Mec-1 WT and 22 µM for Mec-1 Bcl-XL or Mec-1 Mcl-1 cells (Figure 2A,C,E). In contrast, co-incubation with either ABT-737 or ABT-199 did not affect the three cell lines to the same extent. The synergistic effect was clearly detected in Mec-1 WT cells, since ABT-199 and ABT-737 decreased the IC50 to 10 µM and to 5 µM, respectively. In the case of Mec-1 Bcl-XL cells, ABT199 did not improve the IC50, while ABT737 reduced it to 13 µM, while in Mec-1 Mcl-1 cells ABT199 partially lowered the IC50 to 18 µM and ABT737 to 13 µM.

Based on these data, we chose the lowest concentration where ibrutinib alone caused less damage to cells, while still exhibiting a potential combinatory effect with any of the BH3 mimetics. Thus, the concentrations of ibrutinib of 10 μM and ABT-737/ABT-199 of 5 μM were considered as the best choice to test the apoptosis induction in these combination therapies. It should be remarked that ABT-199 is used in the clinic in the management of several hematologic malignancies, and its clinically relevant concentration is between 1 and 2 µM [29], somewhat lower than those used in our study.

Results from the apoptosis induction experiments demonstrated a significant synergistic effect of ibrutinib with both mimetics in Mec-1 WT cells (Figure 2B). Nevertheless, as anticipated by the cell growth assays, this effect was not equally evident in the sublines, especially for Mec-1 Bcl-XL cells (Figure 2D,F). Despite this, we were actually able to detect significant differences for ibrutinib and ABT-737 in Mec-1 Bcl-XL cells and for both ibrutinib and ABT-737/ABT-199 in Mec-1 Mcl-1 cells, suggesting combinatory effects for these drugs, at least to some extent. 

### 3.3. Apoptosis Induction of BH3 Mimetics and Ibrutinib in B-CLL Samples

**Figure 3:**
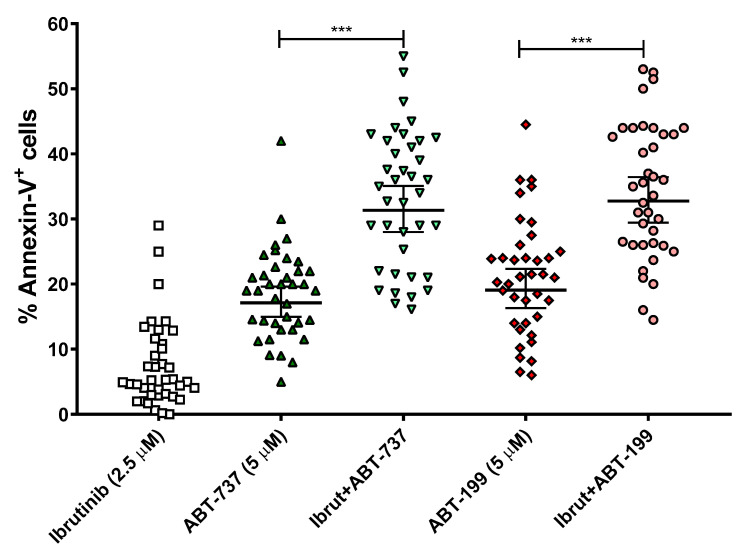
Effect of ABT-737/ABT-199 and/or ibrutinib in cell apoptosis in ex vivo samples from B-CLL patients. Cells were seeded and incubated for 24 h with ABT-737/ABT-199 (5 μM) and/or ibrutinib (2.5 μM). After this, flow cytometry was then employed for apoptosis evaluation. Data are reflected as the mean ± SD of percentage of annexin-V-positive cells. n = 38. ** *p* < 0.01; *** *p* < 0.001.

In addition to the cell death assays in the B-CLL cell lines, it was also intended to test these combination therapies in a total of 38 ex vivo B-CLL samples from patients. The patients used in these experiments were not selected according to their genetic background (e.g., mutations in TP53 or IGVH). With the aim to translate the doses of our treatments to others closer to clinical relevance, we chose to lower the concentrations of ibrutinib to 2.5 μM and those of BH3 mimetics to 5 μM, while maintaining the 24 h incubation. The results are displayed in Figure 3. Ibrutinib was able to achieve a significant combinatory effect with both ABT-737 and ABT-199, while not being toxic on its own. Both ABT-737 and ABT-199 did induce a certain level of cell death by themselves but much less than in combination with ibrutinib.

### 3.4. Assessment of Pro- or Anti-Apoptotic Protein Expression Following Ibrutinib Exposure

Next, we sought to determine whether these combinatory effects could be caused by changes in the expression of Bcl-2 family protein members. Given that the most evident combinatory effects were obtained with Mec-1 WT cells, these experiments were executed using this cell line. Cell extracts were obtained before and after ibrutinib incubation, and Western blots were performed.

**Figure 4:**
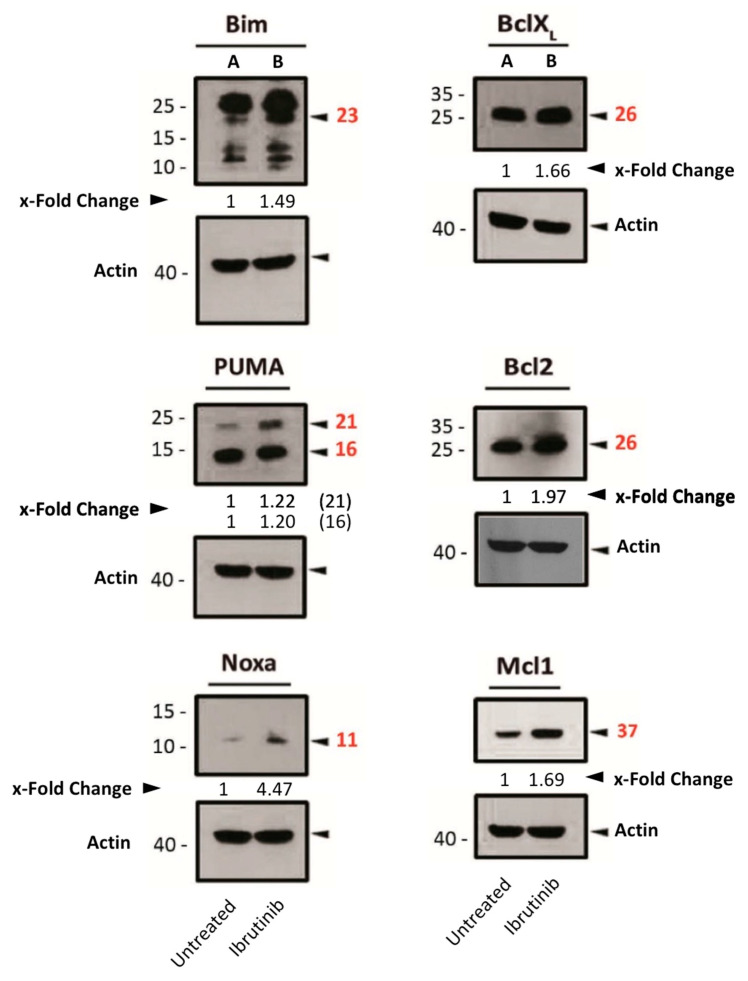
Expression analysis of several pro- or anti-apoptotic proteins in Mec-1 WT after ibrutinib incubation (20 μM, 24 h) by Western blot. Protein extracts from cells were extracted following ibrutinib exposure, samples were loaded into several electrophoresis gels and separation of proteins, transfer onto membranes and Western blots were performed, as indicated in the Material and Methods. Bcl-2, Bcl-XL, Bim, Mcl-1, Noxa, PUMA and actin (as loading control) were analyzed. x-Fold Changes were obtained by calculating the ratio of each protein with its actin control and then dividing again by the corresponding ‘Untreated’ sample. Numbers on the side of the immunoblots correspond to the molecular weight of the proteins expressed in kDa.

After ibrutinib treatment, the expression of both pro-apoptotic (Bim, Noxa and PUMA) and anti-apoptotic (Bcl-2, Bcl-XL and Mcl-1) proteins of the Bcl-2 family was increased (Figure 4). In light of these findings, and based on PUMA’s physiological role in inhibiting anti-apoptotic Bcl-2-family members [30], we hypothesized that the increase in PUMA expression by ibrutinib might be counteracting the other anti-apoptotic proteins by directly binding to them.

Therefore, this possibility was explored by performing immunoprecipitation of PUMA, with antibodies directed either to phosphorylated Bcl-2 (anti- p-Bcl-2 at Ser70) or Bcl-XL (anti-p-Bcl-XL at Ser62).

**Figure 5:**
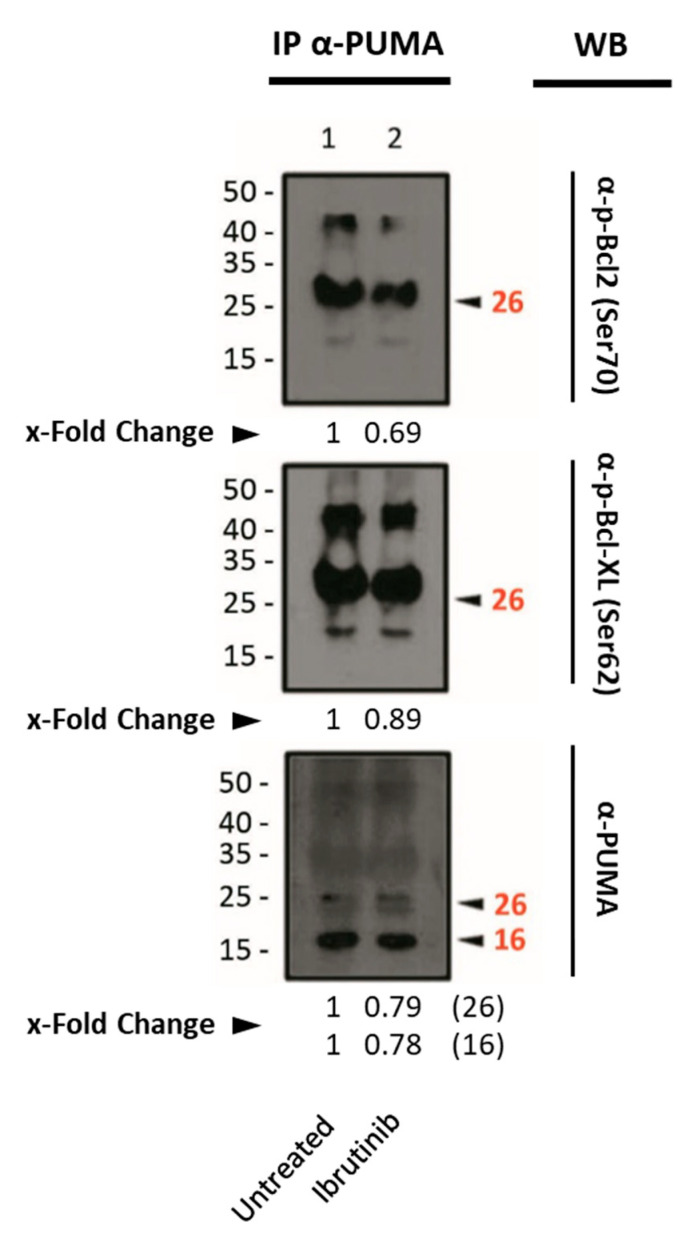
Western blot analysis of the phosphorylated form of the anti-apoptotic proteins Bcl-XL and Bcl-2 from the extracts obtained by immunoprecipitation after Mec-1 WT cell line treatment with ibrutinib (20 μM, 24 h). An antibody against PUMA was used as an immunoprecipitating agent. Ser70 and Ser62 were analyzed as targets for phosphorylation for Bcl-2 and Bcl-XL, respectively. IP: immunoprecipitated phase; WB: Western blot. x-Fold Changes were calculated by dividing the densitometry value obtained for each protein by its corresponding untreated control. Numbers on the side of the immunoblots correspond to the molecular weight of the proteins expressed in kDa.

The immunoprecipitation experiments revealed the interaction between PUMA and Bcl-2 or Bcl-XL. However, we could not detect a clear increase in their interaction upon ibrutinib treatment (Figure 5).

### 3.5. Combination of DCA and Ibrutinib in Cell Growth and Apoptosis in Mec-1 Cell Lines

Metabolic reprogramming is a promising target in B-CLL [31] and has recently been established as a compelling modulator to improve other therapies [32]. For these reasons, apart from exploring the combination ability and mechanism of ibrutinib with either ABT-737 or ABT-199, we also sought to evaluate the ability of DCA—a metabolic drug that forces OXPHOS—to sensitize our Mec-1 cells to the action of ibrutinib. This combinatory treatment was first explored on Mec-1 WT cells. A 10–20 mM dose–response curve with DCA was performed at different incubation times (24–72 h). Then, after 48 or 72 h, ibrutinib was added at a fixed dose of 10 μM for an additional 24 h period. Finally, cell growth inhibition and apoptosis induction were assessed.

**Figure 6:**
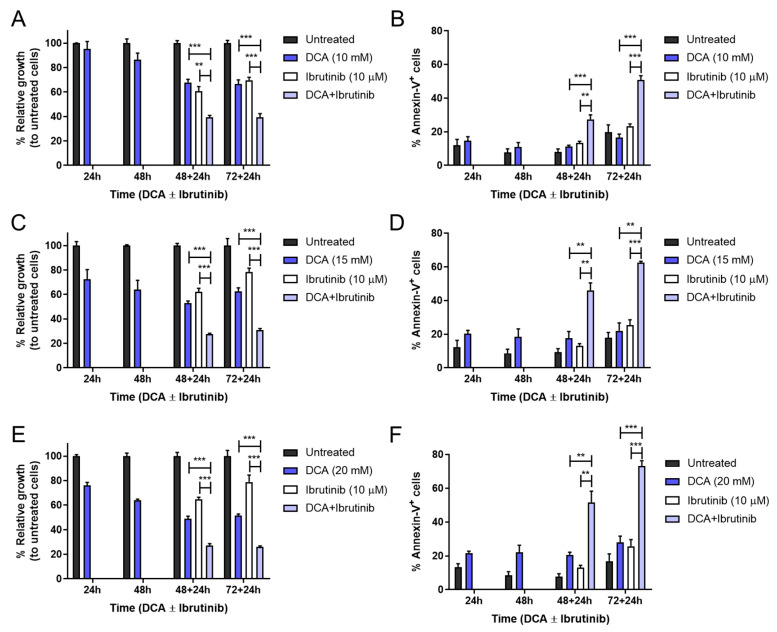
Effect of DCA and/or ibrutinib on cell growth and apoptosis in Mec-1 WT cells. Cells were seeded and preincubated for 24–72 h with DCA (10 mM in (**A**,**B**); 15 mM in (**C**,**D**); 20 mM in (**E**,**F**)). After 48 and 72 h, ibrutinib (10 μM) was added for another 24 h. MTT assays (**A**,**C**,**E**) were then performed for cell growth assessment, while flow cytometry (**B**,**D**,**F**) was employed for apoptosis evaluation. Data from MTT are expressed as the mean ± SD of percentage of relative growth, compared to untreated cells; data from flow cytometry are reflected as the mean ± SD of percentage of annexin-V-positive cells. n = 2–4. ** *p* < 0.01; *** *p* < 0.001.

Significant combinatory effects were obtained for all incubation times and doses of DCA. Analyzing more closely, DCA alone affected Mec-1 WT cell growth inhibition in a dose-dependent manner, albeit no significant effect was detected in apoptosis induction. Regarding DCA + ibrutinib, it was sufficient to induce almost 50 percent cell death and 60 percent growth inhibition at the lower dose of DCA (10 mM) and the longest incubation time (72–24 h, see Figure 6A,B). Furthermore, increasing DCA to 15 or 20 mM improved these parameters to 70 and 60 percent or to 80 and 75 percent, respectively (Figure 6C–F).

Once we examined the effectiveness of this treatment in Mec-1 WT cells, we also sought to evaluate its efficacy in the Mec-1 Bcl-XL and Mec-1 Mcl-1 sublines. Given that we had already observed higher resistance of these cells in the previous treatment, the highest DCA concentration (20 mM) was chosen to perform the experiment, similarly to the previous one.

**Figure 7:**
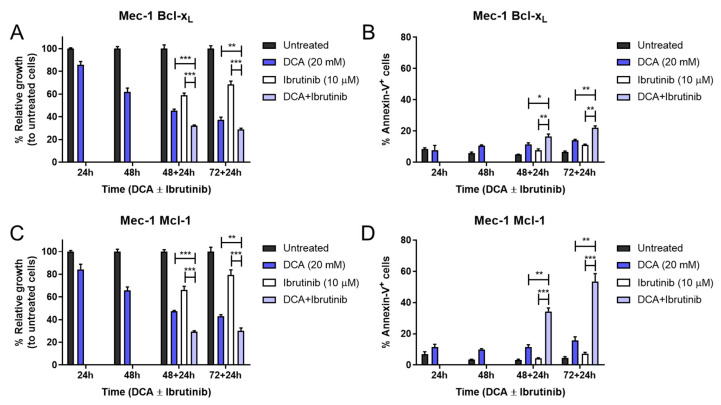
Effect of DCA and/or ibrutinib on cell growth and apoptosis in Mec-1 Bcl-XL (**A**,**B**) and Mec-1 Mcl-1 **(C**,**D**) cells. Cells were seeded and preincubated for 24–72 h with DCA (20 mM). After 48 and 72 h, ibrutinib (10 μM) was added for another 24 h. MTT assays (**A**,**C**) were then performed for cell growth assessment, while flow cytometry (**B**,**D**) was employed for apoptosis evaluation. Data from MTT are expressed as the mean ± SD of percentage of relative growth, compared to untreated cells; data from flow cytometry are reflected as the mean ± SD of percentage of annexin-V-positive cells. n = 2–4. * *p* < 0.01; ** *p* < 0.01; *** *p* < 0.001.

The combination of DCA and ibrutinib was capable of inducing significant increases in cell growth inhibition and cell apoptosis in both cell lines (Figure 7). Despite this, the apoptosis induction of this therapy in Mec-1 Bcl-XL cells (Figure 7B) was notably lower than in Mec-1 WT (Figure 6), but it was almost equivalent for Mec-1 Mcl-1 cells (Figure 7D). This suggests, on one hand, a protecting role of Bcl-XL and, on the other hand, a lack of a protecting role of Mcl-1 in DCA sensitization.

### 3.6. Apoptosis Induction of DCA and Ibrutinib in B-CLL Samples

After confirming this combinatory effect of DCA and ibrutinib in Mec-1 cell lines, we examined its efficacy in ex vivo B-CLL samples from patients. Given that our treatment may last for a maximum of 96 h, and survival of primary ex vivo cells is limited, an additional combinatory time point was implemented, evaluating just a 24 h sensitization with DCA before the addition of ibrutinib.

**Figure 8:**
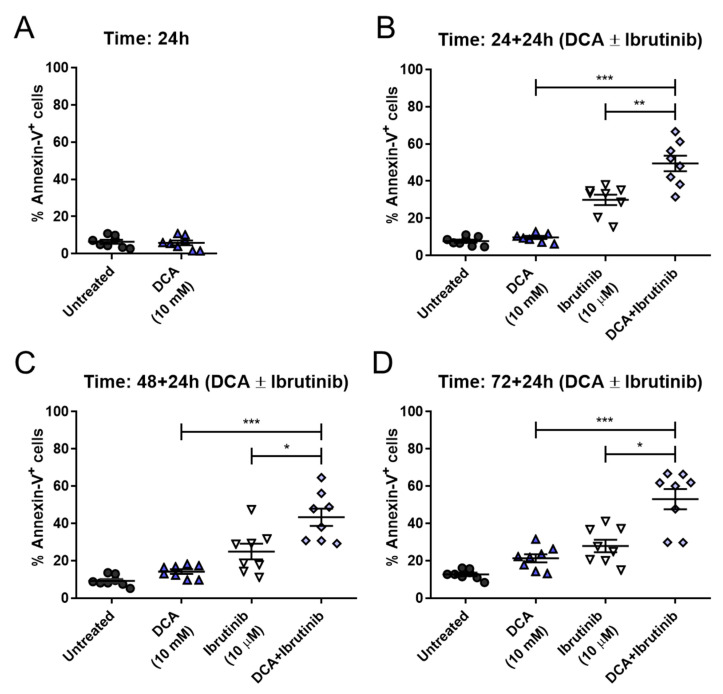
Effect of DCA and/or ibrutinib in cell apoptosis in ex vivo samples from B-CLL patients. Cells were seeded and preincubated for 24 (**A**,**B**), 48 (**C**) or 72 (**D**) hours with DCA (10 mM). After 24 (**B**), 48 (**C**) and 72 (**D**) hours, ibrutinib (10 μM) was added for another 24 h. Flow cytometry was then employed for apoptosis evaluation. Data are reflected as the mean ± SD of percentage of annexin-V-positive cells. n = 8. * *p* < 0.01; ** *p* < 0.01; *** *p* < 0.001.

DCA concentrations achieved in vivo are around 0.5 and 5 mM [33]. The concentrations used in this work are somewhat higher, although the in vivo treatment is extended for several months or years, while our incubations last no longer than 72 h. Regardless, the conditions closer to the in vivo conditions should be those in which the lower 10 mM concentration is used. As shown in Figure 8, pretreatment with 10 mM DCA achieved significant sensitization to the action of ibrutinib at all times tested in cells from B-CLL patients. Cell apoptosis reached around 50 percent at 24 + 24 h, similar to 48 + 24 h or 72 + 24 h. This behavior is clinically relevant, as shorter times for treatment are always more convenient. Furthermore, when comparing these results with those obtained at 10 mM DCA in Mec-1 WT cells, apoptosis achieved by the combination is very similar (see Figure 6B).

Regarding results using DCA at 15 mM, the combinatory therapy also exhibited statistically significant increases in cell apoptosis compared to single treatments. Similarly, the efficacy achieved 60% cell death and was maintained at longer incubation times (Supplementary Figure S1).

The highest cytotoxicity was obtained at the 20 mM DCA concentration (Supplementary Figure S2). In this case, the 24 h sensitization resulted in a cytotoxicity of the combination of around 60%, increasing to 70% as a mean in the 72 h sensitization protocol, although in some patients it reached almost 90%.

### 3.7. Assessment of Pro- or Anti-Apoptotic Proteins Following DCA Exposure

**Figure 9:**
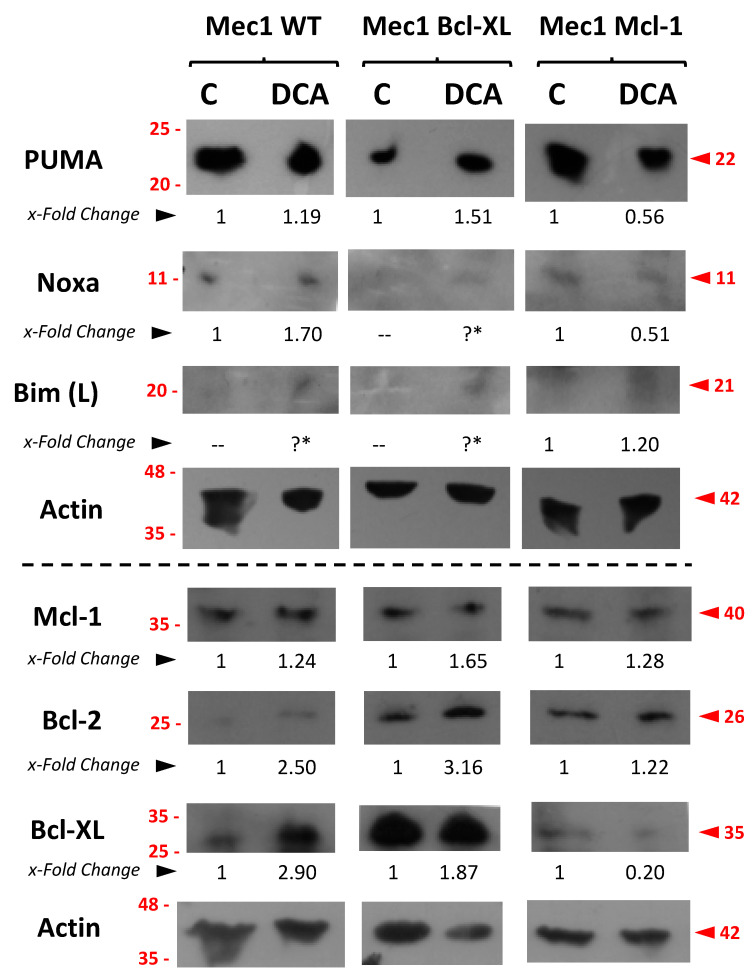
Expression analysis of several pro- or anti-apoptotic proteins in Mec-1 WT, Mec-1 Bcl-XL and Mec-1 Mcl-1 cells, after DCA incubation (5 mM, 72 h) by Western blot. Protein extracts from cells were extracted following DCA exposure, samples were loaded into several electrophoresis gels and separation of proteins, transfer into membranes and Western blots were performed, as indicated in the Material and Methods. PUMA, Noxa, Bim (L, or 23 kDa), Bcl-2, Bcl-XL, Mcl-1 and actin (as loading control) were analyzed. x-Fold Changes were obtained by calculating the ratio of each protein with its actin control and then dividing again by the corresponding ‘Untreated’ sample. Lanes with the “?*” symbol could not be quantified because of the low level of the signal. Numbers on the side of the immunoblots correspond to the molecular weight of the proteins expressed in kDa.

Finally, we sought to explore the possible mechanisms behind DCA sensitization. Therefore, Mec-1 cell lines were incubated with DCA, cell extracts were obtained, and the expression of several pro- and anti-apoptotic proteins that could be involved in this combinatory effect were assessed by several Western blots. Results are displayed in Figure 9.

Regarding pro-apoptotic proteins, we detected a relative increase in the amount of PUMA in Mec-1 and Mec-1 Bcl-XL but not in Mec-1 Mcl1 cells upon DCA treatment. In the case of Bim and Noxa, this could not be explored properly because of their low levels of basal expression (Figure 9). In the case of the anti-apoptotic Bcl-2-family members Bcl-2, Bcl-XL and Mcl-1, a certain increase in their expression was rather observed upon DCA treatment, especially in Mec-1 WT and Mec-1 Bcl-XL cells.

## 4. Discussion

The BCR signaling pathway is instrumental for B-cell development, survival and proliferation. Bruton tyrosine kinase, or BTK, is a downstream enzyme of this pathway that favors the generation of second messengers. Accordingly, B-cell malignancies also rely on this pathway to survive [34]. As already explained in the Introduction, the emergence of the BTK inhibitor ibrutinib redefined therapy for this malignancy [14]. Despite this, several concerns have been raised in recent years regarding its efficacy and secondary effects. First, in several clinical trials, overall survival does not seem to improve compared to other current treatments [16]. Second, ibrutinib has been shown to bind not only to BTK but also to other cysteine-containing kinases as off-targets [35]. In most cases, this does not translate into serious adverse effects [15]. However, one occasional but severe side effect is atrial fibrillation [36]. This and other adverse effects are making physicians to reconsider the dosing quantity and schedule [37].

One approach to solve this problem is the use of second-generation BTK inhibitors, such as acalabrutinib or zanubrutinib. These new pharmacological treatments have already displayed a more specific inhibition of BTK and a safer profile than ibrutinib in their first studies [38]. Another approach is to combine ibrutinib treatment with other therapies; this strategy allows researchers to search for other synergistic approaches, while reducing ibrutinib’s concentration and adverse effects in patients [17].

In addition, there are unmet medical needs in the treatment of B-CLL. For example, the most serious complication during the clinical course of CLL is Richter transformation, which is characterized by the development of an aggressive type of lymphoma [39] and lacks efficient treatment.

In this paper, we decided to focus on the second option, given that ibrutinib still remains as the most used BTK inhibitor in this malignancy and combinatory treatments open the possibilities for multiple approaches.

The combination of ibrutinib and BH3 mimetics, such as ABT-737 and especially ABT-199, has already been proposed and studied before [40]. In fact, ibrutinib and ABT-199 have reached phase II in a clinical trial so far [41]. Mechanistically, ABT-199 inhibits Bcl-2, ABT-737 inhibits both Bcl-2 and Bcl-XL and ibrutinib blocks BTK (among other kinases, as already commented). The rationale behind combining these two classes of drugs has been that both anti-apoptotic Bcl-2-family proteins and BCR signaling are usually overexpressed or overactivated, respectively, in B-CLL. To better analyze this combination, we have generated two different sublines from the B-CLL cell line Mec-1 that overexpress either Bcl-XL or Mcl-1.

A synergistic effect of the combination was obtained, especially in Mec-1 WT cells. In general, both Mec-1 Bcl-XL and Mec-1 Mcl-1 tolerated BH3 mimetics better than Mec-1 WT cells. This result was expected given that they overexpress these anti-apoptotic Bcl-2 family members. However, the combination of ibrutinib with the BH3 mimetics also returned positive results in the case of the sublines. Importantly, the positive combinatory effect was also demonstrated in a cohort of 38 B-CLL patients.

Regarding the mechanism of action of ibrutinib-induced cell death, we noticed that there was almost no difference in its IC50 against Mec-1 WT or against Mec-1 Bcl-XL or Mec Mcl-1 cells. However, the combination with the BH3 mimetics showed synergistic effects in the three cell lines, although cell death in Mec-1 Bcl-XL and Mec-1 Mcl-1 was lower than that observed in Mec-1 WT cells. Therefore, its synergistic effect with the BH3 mimetics suggests a participation of the mitochondrial apoptotic pathway. Hence, we evaluated the expression of pro-apoptotic or anti-apoptotic members of the Bcl-2 family after ibrutinib treatment. A rise in the expression of Bim, PUMA and Noxa was detected. At the same time, the anti-apoptotic proteins Bcl-2, Bcl-XL and Mcl-1 also increased upon ibrutinib incubation. Given that the higher expression among the pro-apoptotic proteins was that of PUMA, we performed immunoprecipitation of this protein with phosphorylated Bcl-2 or Bcl-XL, with which PUMA competes and counteracts. However, a clear increase in the interaction between PUMA and those proteins upon ibrutinib incubation could not be detected. In relation to these results, it has already been described that patients with resistant B-CLL previously treated with ibrutinib often possess higher expression of these anti-apoptotic proteins [42].

The other approach explored in this study was related to the ability of the metabolic inhibitor dichloroacetate (DCA) to sensitize our cell models to the action of ibrutinib, for the reasons outlined in the Introduction. In this case, all cell lines displayed a significant increase in cell growth inhibition and cell apoptosis with the combined therapy compared with the drugs alone. Again, Mec-1 Bcl-XL cells were the ones that manifested the highest resistance for all treatments. Focusing on the combinatory effect, it can be deduced that there must be a mechanism by which Bcl-XL limits DCA-induced sensitization to ibrutinib. Interestingly, DCA increased Bcl-XL expression in Mec-1 WT cells, but this fact did not dampen the positive combinatory effect on these cells. In line with these results, other publications have already registered a rise in Bcl-XL expression after DCA incubation in several tumor types, such as lung adenocarcinoma, colorectal cancer [25] and oral squamous cell carcinoma [43], and, still, that did not stop the therapies from working. Other groups, rather than an increase, have detected no changes in Bcl-XL upon DCA treatment [44,45]; nevertheless, a down-modulation of its expression was registered. As a general conclusion, it seems clear that Bcl-XL protects from DCA sensitization to cell death, as demonstrated in Mec-1 Bcl-XL cells, indicating a participation of the mitochondrial apoptotic pathway machinery in the sensitization. This correlates with a relative increase in PUMA expression induced by DCA, although it also induces a rise in the pro-apoptotic members of the Bcl-2 family. DCA forces OXPHOS in cells and some of the well-known side effects of it are mitochondrial membrane depolarization (MMP) and reactive oxygen species (ROS) generation [46]. Conversely, one of the ways by which Bcl-XL promotes survival in cells is precisely by regulating membrane potential and ROS homeostasis in mitochondria [47].

This combined therapy also demonstrated a synergic effect in B-CLL cells in ex vivo samples. Data from patient-derived tumors were similar to those obtained in cell lines, and also at the shortest incubation times, a good indication for the possible clinical use of this combination.

Beyond the mechanistic intricacies behind these combined therapies, it would be of interest to test their efficacy in in vivo experiments. Especially, the combination of DCA and ibrutinib, since the ibrutinib + BH3 mimetics combination has already been tested. In addition to this, it could be intriguing to assess whether these combined therapies are also effective in other B-cell malignancies and which mechanism they follow.

## 5. Conclusions

In this manuscript, we have demonstrated the effectiveness of combining ibrutinib with different approaches: BH3 mimetics (ABT-737 and ABT-199) and DCA, in in vitro and also ex vivo B-CLL samples. Mechanistically, our findings described a role of Bcl-XL in the resistance of B-CLL cells to these therapies. Regarding pro-apoptotic protein expression, although there is a tendency to increase the expression of PUMA in both settings, we could not demonstrate a clear implication of this protein in the mechanism of action of ibrutinib or in the sensitization to this drug by DCA. This study provides new insights behind already existing and new alternatives for ibrutinib-based therapies.

## Data Availability

Data are available from the corresponding author upon reasonable request.

## Supplementary Materials

The following supporting information can be downloaded at https://www.mdpi.com/article/10.3390/cells14171343/s1. Figure S1: Effect of DCA and/or ibrutinib on cell apoptosis in ex vivo samples from B-CLL patients (DCA at 15 mM). Figure S2: Effect of DCA and/or ibrutinib on cell apoptosis in ex vivo samples from B-CLL patients (DCA at 20 mM).
