# Supplementary material for: The Combination of Ibrutinib with BH3 Mimetics or Dichloroacetate Is Effective in B-CLL
Source: Cells. 2025 Aug 29;14(17):1343. doi: 10.3390/cells14171343 (PMC12427631; doi:10.3390/cells14171343)
Supplement: Supplementary file 1 [file cells-14-01343-s001.zip › cells-3791618-supplementary.pdf]

Supplemental Material

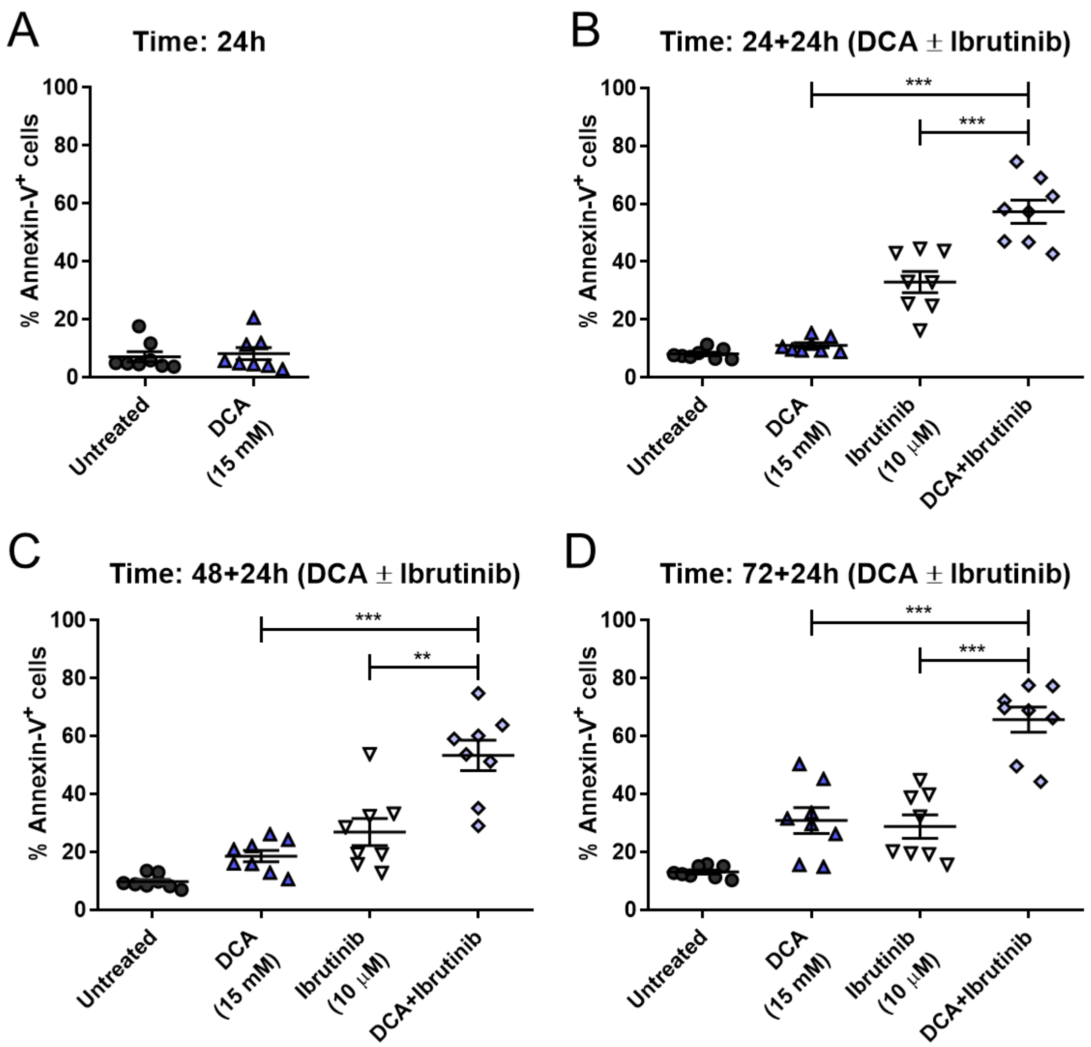

Suppl Fig 1. Effect of DCA and/or ibrutinib in cell apoptosis in *ex vivo* samples from B-CLL patients. Cells were seeded and preincubated for 24 (A, B), 48 (C) or 72 (D) hours with DCA (15 mM). After 24 (B), 48 (C) and 72 (D) hours, ibrutinib (10 µM) was added for another 24 hours. Flow cytometry was then employed for apoptosis evaluation. Data are reflected as the mean +/- SD of percentage of annexin-V positive cells. n=8. *p<0.01; **p<0.01; ***p<0.001.

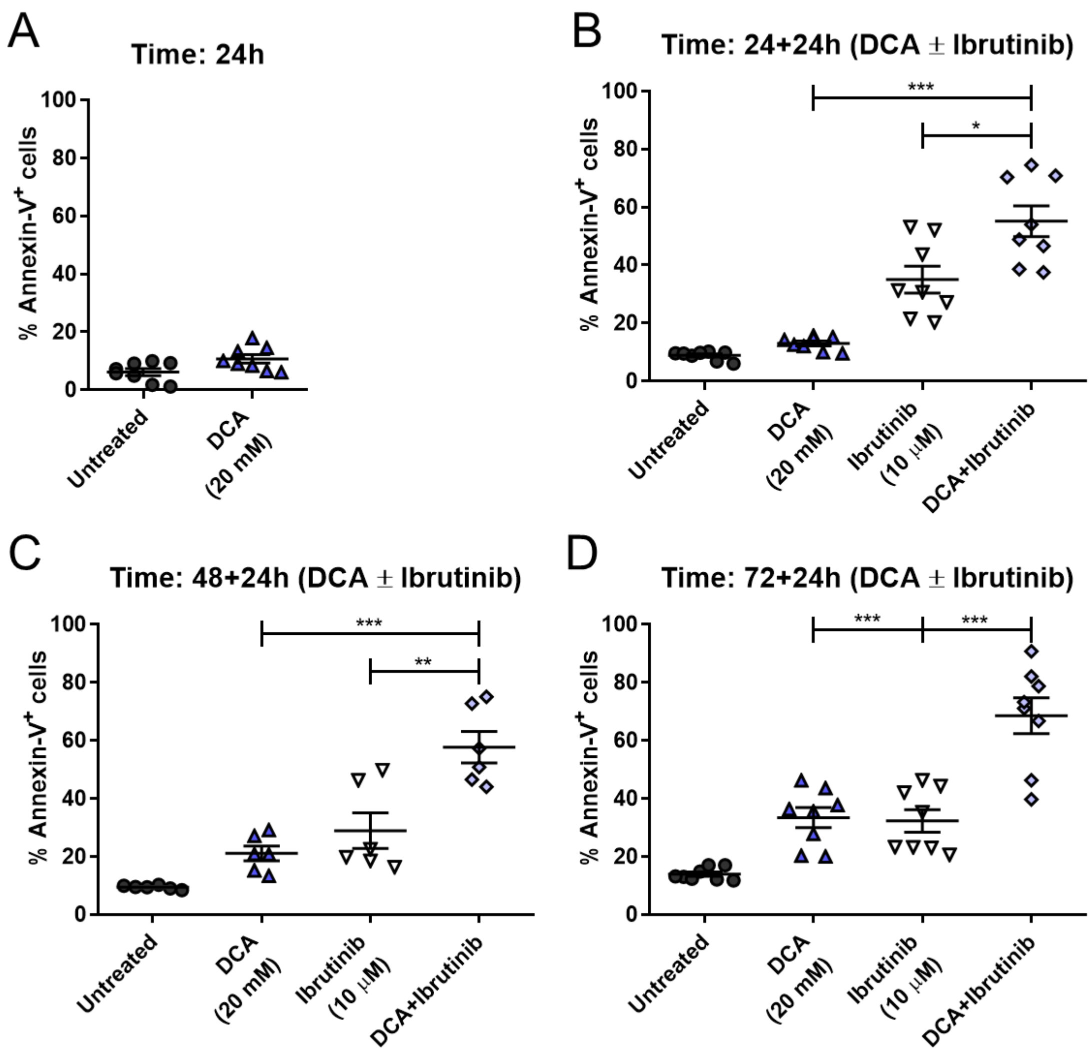

Suppl Fig 2. Effect of DCA and/or ibrutinib in cell apoptosis in *ex vivo* samples from B-CLL patients. Cells were seeded and preincubated for 24 (A, B), 48 (C) or 72 (D) hours with DCA (20 mM). After 24 (B), 48 (C) and 72 (D) hours, ibrutinib (10 µM) was added for another 24 hours. Flow cytometry was then employed for apoptosis evaluation. Data are reflected as the mean +/- SD of percentage of annexin-V positive cells. n=8. *p<0.01; **p<0.01; ***p<0.001.
